# Upregulation of microRNA 142-3p in the peripheral blood and urinary cells of kidney transplant recipients with post-transplant graft dysfunction

**DOI:** 10.1590/1414-431X20175533

**Published:** 2017-04-03

**Authors:** T.D. Domenico, G. Joelsons, R.M. Montenegro, R.C. Manfro

**Affiliations:** 1 Programa de Pós-Graduação em Medicina: Ciências Médicas, Faculdade de Medicina, Universidade Federal do Rio Grande do Sul, Porto Alegre, RS, Brasil; 2 Unidade de Transplante Renal, Serviço de Nefrologia, Hospital de Clínicas de Porto Alegre, Porto Alegre, RS, Brasil

**Keywords:** microRNA, Graft dysfunction, Renal transplantation, Gene expression, Biomarker

## Abstract

We analyzed microRNA (miR)-142-3p expression in leucocytes of the peripheral blood and urinary sediment cell samples obtained from kidney transplant recipients who developed graft dysfunction. Forty-one kidney transplant recipients with kidney graft dysfunction and 8 stable patients were included in the study. The groups were divided according to histological analysis into acute rejection group (n=23), acute tubular necrosis group (n=18) and stable patients group used as a control for gene expression (n=8). Percutaneous biopsies were performed and peripheral blood samples and urine samples were obtained. miR-142-3p was analyzed by real-time polymerase chain reaction. The group of patients with acute tubular necrosis presented significantly higher expressions in peripheral blood (P<0.05) and urine (P<0.001) compared to the stable patients group. Also, in the peripheral blood, miR-142-3p expression was significantly higher in the acute tubular necrosis group compared to the acute rejection group (P<0.05). Urine samples of the acute rejection group presented higher expression compared to the stable patients group (P<0.001) but the difference between acute tubular necrosis and acute rejection groups was not significant in the urinary analyzes (P=0.079). miR-142-3p expression has a distinct pattern of expression in the setting of post-operative acute tubular necrosis after kidney transplantation and may potentially be used as a non-invasive biomarker for renal graft dysfunction.

## Introduction

Renal transplantation is currently the treatment of choice for many patients with end-stage kidney disease providing significant improvements in life quality and expectancy (1–3). However, despite the advances in the field, acute rejection (AR) remains a significant cause of graft damage and can lead to its failure (4–6). Also, as a result of ischemia and reperfusion injuries, post-transplant acute tubular necrosis (ATN) occurs frequently post-operatively and creates difficulties in the diagnosis of AR and of other causes of graft dysfunction (7).

The diagnosis of post-operative ATN requires a graft biopsy and other conditions, including mainly AR, infections, and drug toxicities, which may occur concomitantly. The ATN diagnosis may be complicated by the presence of a non-specific tubulo-interstitial cell infiltrate and a lack of synchrony between structure and function is frequently observed in this setting (8,9). Additionally, the incidence of AR during the period of delayed graft function (DGF) has been shown to be higher. A meta-analysis of 34 studies from 1988 through 2007 concluded that patients with DGF had a 49% pooled incidence of acute rejection compared to 35% incidence in non-DGF patients (10). The kidney biopsy, however, has a number of shortcomings including its invasiveness, risk, sampling error, poor interpretation reproducibility and high costs. As a consequence, the development of non-invasive biomarkers for the different causes of graft injury is highly desired for aiding clinical practice.

MicroRNAs (miRNAs) are small fragments of non-coding DNA that are highly conserved across species (11). A single miRNA may negatively regulate hundreds of messenger RNAs (mRNAs). In turn, the expression of one mRNA may be regulated by several miRNAs acting in tandem. These small RNA fragments control processes such as cell development, proliferation, differentiation, apoptosis and metabolism, and their deregulation may lead to derangement and suppression of genes that play a role in intracellular cascade signaling, leading to disease onset or progression (12–15). miRNAs are actively involved in the regulation and development of cells of the immune system. It has been recently demonstrated that specific miRNAs have a significant impact on B and T-cell differentiation and on a variety of other processes related to innate and adaptive immunity, including inflammation, signaling, cytokine production, regulatory T-cells (Treg) function, and antigen presentation, as well as a role on hypoxia- and reperfusion-related injuries (13 [Bibr B14],^16^).

MicroRNA-142-3p (miR-142-3p) is directly involved in the suppression of immune system functions, particularly in the differentiation and suppression of CD4+CD25+ Tregs (13,17,18). It also plays a role in the stimulation of transforming growth factor-β (TGF-β) (19) and acts as a cyclic AMP regulator (18,19). Also, miR-142-3p has considerable specificity for hematopoietic lineages whose components infiltrate allografts both in rejection and in cell necrosis processes (20,21), and elevated levels of this miRNA may suggest the presence of an inflammatory response (20). In the present study, we analyzed miR-142-3p expression in the peripheral blood and urinary sediment cells of kidney transplant recipients with graft dysfunction. Our aim was to verify whether this miRNA would be differentially expressed in non-invasive samples of kidney transplant recipients with ATN or with acute rejection.

## Patients and Methods

### Patients

Forty-one kidney transplant recipients with kidney graft dysfunction and 8 stable patients were included in the study. All patients were on immunosuppressive drug therapy with a combination of corticosteroids, sodium mycophenolate, and tacrolimus. Induction with either Basiliximab or rabbit anti-thymocyte globulin was used for all deceased-donor graft recipients and living-donor graft recipients at higher risk of rejection. Dysfunctional grafts were submitted to indication biopsies and the patients were classified into three groups, acute rejection (AR) 23 patients, acute tubular necrosis (ATN) 18 patients, according to the Banff 07 classification (22). A group of 8 stable patients who had a normal protocol biopsy at three months after transplantation, was used as a control group (STA).

All patients provided written informed consent for participating. The study was approved by the hospital's research Ethics Committee.

### Methods

Percutaneous biopsies were performed under real-time ultrasound guidance, using a semi-automatic biopsy gun with a 16G needle. Peripheral blood samples and urine samples were obtained immediately before the renal biopsy. For cell separation, both sample types were rinsed and processed so as to concentrate peripheral blood mononuclear cells or to obtain urinary sediment cells. For the EDTA-blood samples, the erythrocyte-lysing buffer EL (Qiagen Inc., USA) was used and the resulting cell concentrate was flash frozen using liquid nitrogen and stored at -80°C. For urine samples, the entire volume was centrifuged and the sediment of the tubes resuspended in 750 µL of phosphate-buffered saline and after another centrifugation the supernatant was discarded and the cell sediment was flash frozen using liquid nitrogen and stored at -80°C.

miRNAs were extracted from samples using the *mir*Vana^™^ PARIS^™^ commercial kit (Ambion^®^, Life Technologies Corporation, USA). Briefly, cell concentrate/sediment was dissolved and tissue was fragmented with 500 µL of buffer in a dispersing machine (Ultra-Turrax T 10 basic - IKA, Brazil) and eluted in 60 µL of water for injection preheated to 95°C, in accordance with manufacturer's instructions.

Residual contaminating DNA was removed using the DNA-free^™^ kit (Applied Biosystems^®^, Life Technologies Corporation, USA) and the purified samples were stored at -80°C until the next stage. The concentration of extracted miRNA was quantified in a full-spectrum spectrophotometer (220–750 nm) with sample retention technology (Nanodrop 1000, Thermo Fischer Scientific, USA). The nucleic acid concentration is reported in ng/µL, based on absorbance at 260 nm, and purity was calculated on the basis of the A260/280 and A260/230 ratios. A ratio of approximately 2.0 is generally accepted as "pure" RNA. Samples were considered viable if they had a concentration of at least 2 ng/µL. All samples with a higher concentration were diluted to this concentration in a 50-µL volume of nuclease-free water.

Specific miR-142-3p TaqMan primers (Applied Biosystems" catalog number 4427975/000464) were used for real-time reverse transcription polymerase chain reaction (RT-PCR). Sample normalization was performed with a synthetic exogenous control Cel-miR-39 from *C. elegans* (Qiagen, catalog number MSY0000010), which was spiked into samples before the reverse transcription stage in a 0.5 µL volume at a 50 pM concentration. Complementary DNA (cDNA) synthesis was carried out with the TaqMan MicroRNA RT kit (Applied Biosystems^®^) as per manufacturer instructions, then stored at -20°C until the time of RT-PCR, which consisted of the amplification of 2 µL cDNA using 5 µL of TaqMan Universal PCR Master Mix (Applied Biosystems^®^), 0.5 µL of specific primers, and 2.5 µL of nuclease-free water, in a final reaction volume of 10 µL. The reaction was run on an ABI Prism 7000 system (Applied Biosystems^®^). Samples were incubated for 10 min at 95°C, followed by 40 cycles at 95°C for 15 s and 60°C for 1 min. The cycle threshold (Ct) was calculated automatically by the machine software. miRNA expression was quantified using the 2^-ΔΔCt^ method, as described by Livak and Schmittgen (23).

### Statistical analyses

Asymmetrically distributed variables are reported as medians and interquartile ranges, whereas symmetrically distributed variables are reported as means±SD. The Kruskal-Wallis and Mann-Whitney U tests were used for paired-samples analysis of variance and for between-group analysis. Qualitative data are reported as absolute and relative counts, and the chi-square or Fisher's exact tests were used for between-group analyses. All tests were two-tailed, and a P-value <0.05 was defined as statistically significant. All analyses were carried out in PASW Statistics 18 (SPSS Inc., USA).

## Results

Demographic data of the patients are reported in Table 1. DGF occurred differently among groups and the highest incidence was observed in the ATN group, as expected (P<0.05). DGF was present in the ATN group, and some patients in the AR and STA group presented previous DGF. Induction with polyclonal anti-T cell antibodies was more frequent in the ATN group as well (P<0.05). Lower serum creatinine at biopsy (P=0.07) and longer time between transplantation and biopsy (P=0.06) were observed in the STA group with borderline statistical significance.

**Table 1:**
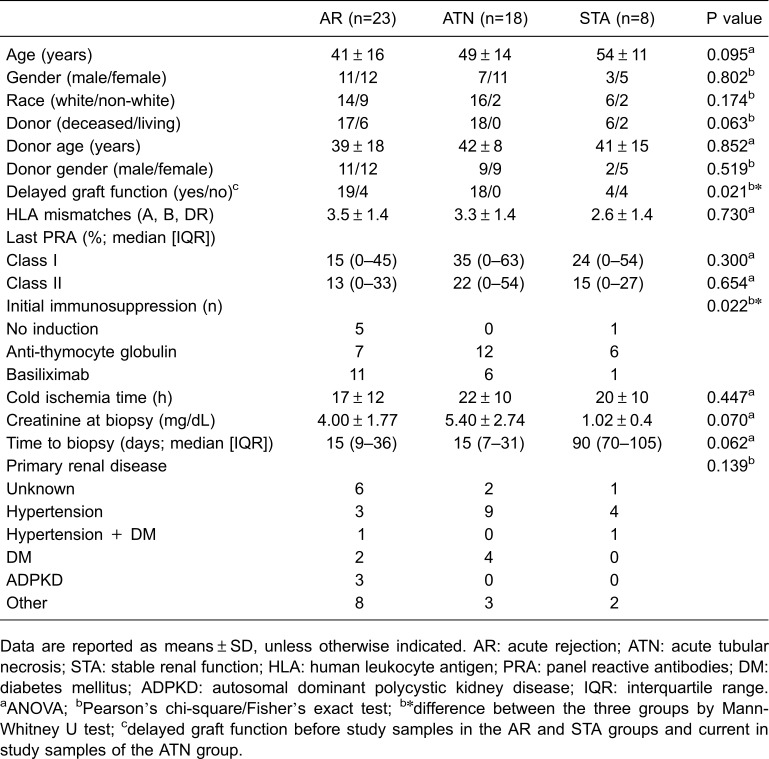
Demographic data of the groups.

**Figure 1:**
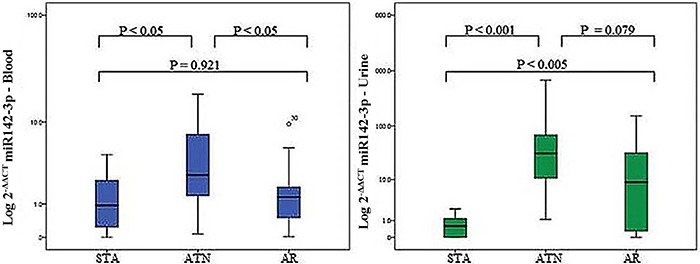
Logarithmic transformation of microRNA 142-3p expression in the peripheral blood and urine of renal transplanted recipients. STA: stable group of patients; ATN: group of patients with acute tubular necrosis; AR: group of patients with acute rejection. Data are reported as medians and interquartile range. Statistical analysis was performed with Mann-Whitney *U* test.

miR-142-3p expression levels are shown in Figure 1. In the peripheral blood analysis, substantially higher expressions were found in the comparisons of the ATN with the STA group (P<0.05) and with the AR group (P<0.05) and no difference was found in the comparison between the STA and AR groups (P=0.921). The analysis of the miR-142-3p from the urinary sediment cells showed significantly higher expression in the ATN group (P<0.001) and AR group (P<0.005) in comparison with the STA group. Also, the expression was higher, with a borderline significance level, in the ATN group compared with the AR group (P=0.079).

**Figure 2:**
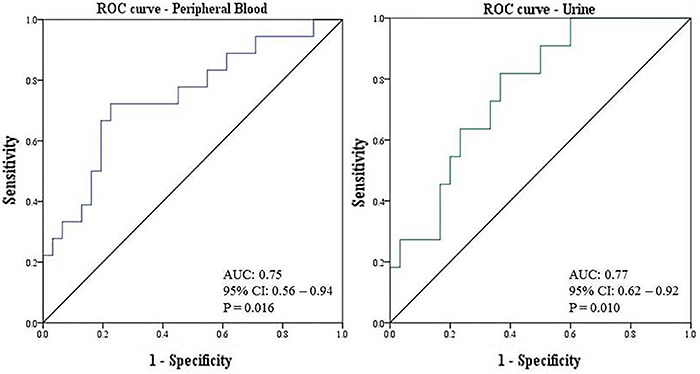
Receiver operating characteristic curves (ROC) of miR-142-3p expressions in the peripheral blood and urine of renal transplanted recipients. AUC: area under the curve; CI: confidence interval.

Receiver operating characteristics (ROC) curves were built for the assessment of the diagnostic parameters for ATN and are shown in Figure 2. In the peripheral blood analysis, the area under the curve (AUC) was 0.75 (95%CI=0.56-0.94; P=0.016). In the urinary cells analysis, the AUC was 0.77 (95%CI=0.62-0.92, P=0.010). The cut-off selected for the blood samples analysis was 1.59 and the resulting parameters were sensitivity=75%; specificity=63%; positive predictive value=81%, and a negative predictive value=50% (P<0.05). For the urinary samples analysis, the cut-off selected was 2.03 and the resulting parameters were sensitivity=92%; specificity=87%; positive predictive value=90% and a negative predictive value=87% (P<0.001).

## Discussion

The understanding of the molecular basis of allograft injuries remains an important and incompletely solved issue in organ transplantation. The molecular mechanisms appear to be highly regulated and the interplay between mRNAs and miRNAs are probably crucial (24). Moreover, the discovery of noninvasive biomarkers that reflect accurately graft-related events is an unmet need in the clinical practice of organ transplantation, since the current available methods are either invasive or lack accuracy. In the present study, we evaluated the expression of miR-142-3p as a biomarker of injury in the immediate post-renal transplantation period and found that its expression was significantly increased in the peripheral blood and in the urinary sedimentary cells of patients with kidney grafts undergoing post-transplant ATN.

In the last decade, miRNAs were studied as biomarkers in renal transplantation (25). Over time, a number of reports have suggested their usefulness as potential noninvasive biomarkers of acute rejection in the peripheral blood and in the urine (26). Also, miRNAs expression has been studied in other post-transplant conditions, such as chronic graft dysfunction, acute pyelonephritis, BK virus nephropathy and operational tolerance (27–29). In many of these studies, specific miRNAs were uncovered by analysis in high throughput platforms and validated by PCR techniques. The above studies lead to the notion that these molecules might become useful biomarkers of specific clinical conditions after organ transplantation.

Cloning studies allowed the determination of miRNAs from opposite arms of the hairpin precursor. As for miR-142, it is processed into two mature miRNAs: miR-142-3p and miR-142-5p. Merkerova and colleagues examined their expression in hematopoietic cell lineages and found that miR-142-3p was approximately 10-fold more expressed than miR-142-5p in this compartment, whose components infiltrate allografts both in rejection and in cell necrotic events (30). Therefore, its increased expression in these conditions might be expected. miR-142-3p expression has been demonstrated in normal human T cells and granulocytes and weak levels of expression occur in monocytes and B cells. Increased levels of expression suggest inflammatory processes within allografts that could be due to either rejection or necrosis (20). This miRNA has been reported to be more expressed in naive T cells than in differentiated Th1 and Th2 cells (31). However, it has been shown that the transcription factor FoxP3 is one of the mediators of transcriptional repression of miR-142-3p and increases in this messenger RNA expression are associated with acute rejection, and thus can lead to decrease in the expression of the miR-142-3p (3,25,32). In support, we found in the present study significant increases of this miRNA in the peripheral blood and urine of kidney graft patients undergoing ATN compared to those with stable grafts and AR, suggesting a more relevant role for the cell necrosis processes in the increase of this miRNA.

Previous research has reported on the miR-142-3p expression on graft tissue and non-invasive samples (peripheral blood or urine) from kidney transplant recipients. Danger et al. (33) verified the expression of miR-142-5p (5′ arm of miR-142) in the peripheral blood and renal graft tissue of patients with chronic antibody-mediated rejection and found it to be over expressed in comparison with patients with stable graft function. The authors reinforced the participation of this miRNA in immunological disorders, and similar with our findings, did not find a significant increase in the expression of this biomarker in patients with acute rejection (33).

Scian et al. (34) evaluated miRNAs expression in allograft tissue and paired urine samples of kidney transplant recipients with chronic allograft dysfunction and interstitial fibrosis and tubular atrophy (IF/TA). They reported that the miR-142-3p expression is increased in patients with IF/TA, both in renal graft tissue and urine and suggested the potential use of miRNAs as noninvasive markers of IF/TA and for monitoring graft function. Ben-Dov et al. (27) found that this miRNA is overexpressed in biopsies of patients with IF/TA in comparison with normal allograft biopsies. Maluf et al. (35) described that in the early period after kidney transplantation urinary miR-142-3p, along with other four miRNAs, were differentially expressed in the group of patients that developed IF/TA in the transplant course. Interestingly, overexpression was detectable before histological allograft injury was evident suggesting that miRNAs are potential biomarkers for monitoring graft function in the anticipation of progression to chronic graft dysfunction. Samples of patients with IF/TA were not included in our study therefore we could not confirm or deny the above findings. However, IF/TA may often be related to inflammatory injuries and increased expression of miR-142-3p might therefore be expected.

Anglicheau et al. (13) demonstrated elevated intragraft miRNA expression in both stable allografts and those undergoing acute rejection. The authors found that 17 miRNAs, including miR-142-5p, which is functionally related to miR-142-3p, could individually distinguish biopsy samples from allografts with acute rejection from those without rejection. Soltaninejad and colleagues analyzed the expression levels of miR-142-3p, miR-142-5p and others in paired biopsy and peripheral blood mononuclear cell samples of renal allograft recipients with acute T-cell mediated rejection, comparing with normal allografts. These authors found elevated levels of miR-142-3p in blood samples of acute T-cell mediated rejection and reported that analyzes of miR-142-3p expression in the peripheral blood could predict acute T-cell mediated rejection. Interestingly a correlation could not be observed between miR-142-3p expressions in biopsy tissue and peripheral blood mononuclear cell (36). Inflammatory infiltrates are present in acute cellular rejection and ATN, therefore an increase in miR-142-3p might be expected in both situations. Neither of the above studies included patients with ATN; thus, comparisons with the present study are difficult to make and further research may be necessary to clarify these findings.

Finally, studies in which miR-142-3p was tested in drug-free tolerant kidney transplant recipients identified this molecule as possibly involved in tolerance mechanisms, probably related to the negative regulation of TGF-β signaling. Danger et al. reported an increased expression of miR-142-3p in B cells purified from operationally tolerant kidney graft recipients in comparison with stable graft recipients under immunosuppressive therapy (19). It has been suggested that this miRNA might be a promising predictor of patients eligible for immunosuppression weaning or withdrawal (19,37).

In conclusion, the stability of miRNAs, together with the possibility of being sequentially obtained from biological fluids, makes their study appealing as potential biomarkers for allograft related injuries. However, the isolation and quantification of miRNAs is a technically time-consuming and expensive process and further optimization of these procedures may be required before their routine application in clinical practice (38). A profound understanding of the complex interplay between miRNAs and mRNAs in the graft response may lead to a better understanding of pathophysiology and mechanisms of graft injuries (24,39).

In this research, we described that miR-142-3p is over-expressed in non-invasive samples of kidney transplant recipients with ATN and may became an useful biomarker of such condition. However, appropriate validation of the molecular approaches in adequately designed longitudinal studies is necessary before clinical applicability.
